# 4-Amino-3,5-di­chloro­pyridinium 3-hy­droxy­pico­linate monohydrate

**DOI:** 10.1107/S2414314623008210

**Published:** 2023-09-22

**Authors:** Agalya Ashokan, Jayasudha Nehru, Nandhini Chakkarapani, Themmila Khamrang, Savaridasan Jose Kavitha, Venkatachalam Rajakannan, Madhukar Hemamalini

**Affiliations:** a Department of Chemistry, Mother Teresa Women’s University, Kodaikanal, Tamil Nadu, India; b Department of Crystallography and Biophysics, University of Madras, Guindy Campus, Chennai-600 025, Tamil Nadu, India; c Assistant Professor, Department of Chemistry, DM College of Science, Dhanamanjuri University, Imphal, Manipur-795 001, India; University of Aberdeen, United Kingdom

**Keywords:** crystal structure, hydrogen bonding, hydrated salt

## Abstract

In the title hydrated salt, C_5_H_5_Cl_2_N_2_^+^·C_6_H_4_NO_3_^−^·H_2_O, the pyridine N atom of the cation is protonated and an intra­molecular O—H⋯O hydrogen bond is observed in the anion, which generates an *S*(6) ring. The crystal packing features N—H⋯N, O—H⋯O, N—H⋯O, C—H⋯Cl and C—H⋯O hydrogen bonds, which generate a three-dimensional network.

## Abstract 2

The title hydrated salt features a dense array of hydrogen bonds, forming a three-dimensional network.

## Graphical abstract

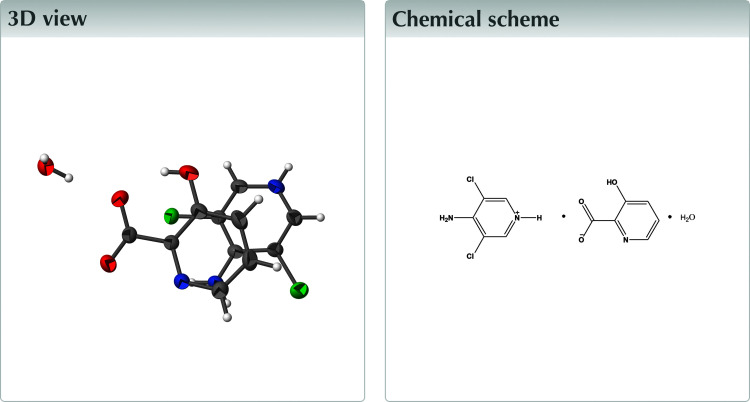

## Structure description

4-Amino­pyridine and its derivatives are used clinically to treat Lambert–Eaton myasthenic syndrome and multiple sclerosis because they block potassium channels, which prolongs action potentials and increases transmitter release at the neuromuscular junction (Judge & Bever, 2006 ▸). Picolinic acid, which contains N and O donors, has attracted much attention for the design and synthesis of self-assembling systems (*e.g.*, Steiner, 2002 ▸). In this regard, 3-hy­droxy­picolinic acid is of inter­est because it can be used as a neutral ligand or, depending on the pH value, as an anionic or cationic ligand. In addition, due to the arrangement of its functional groups, it can act as a monodentate or bidentate ligand, which allows it to form five- or six-membered chelate rings. As part of our work in this area, we now report the synthesis and structure of the title hydrated mol­ecular salt.

**Figure 1:**
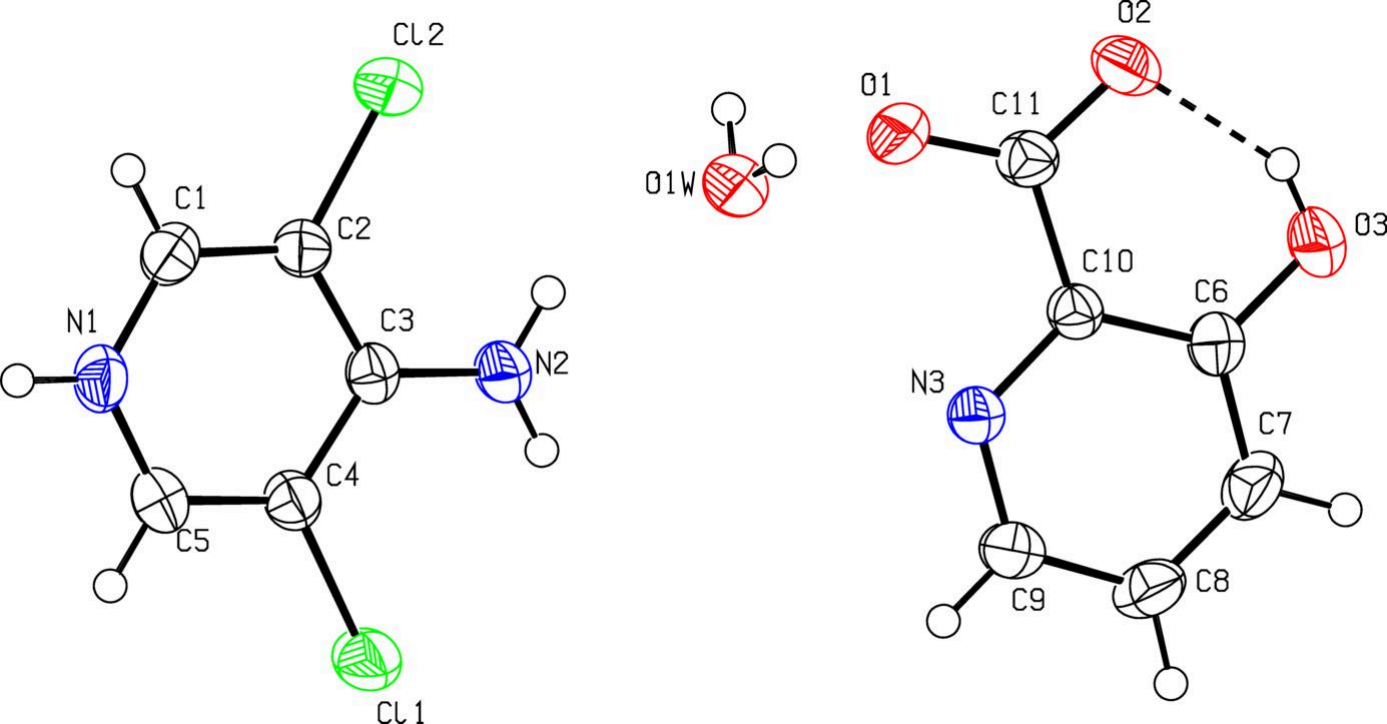
The mol­ecular structure of the title compound showing 50% displacement ellipsoids. The intra­molecular hydrogen bond is shown with dashed lines.

**Figure 2:**
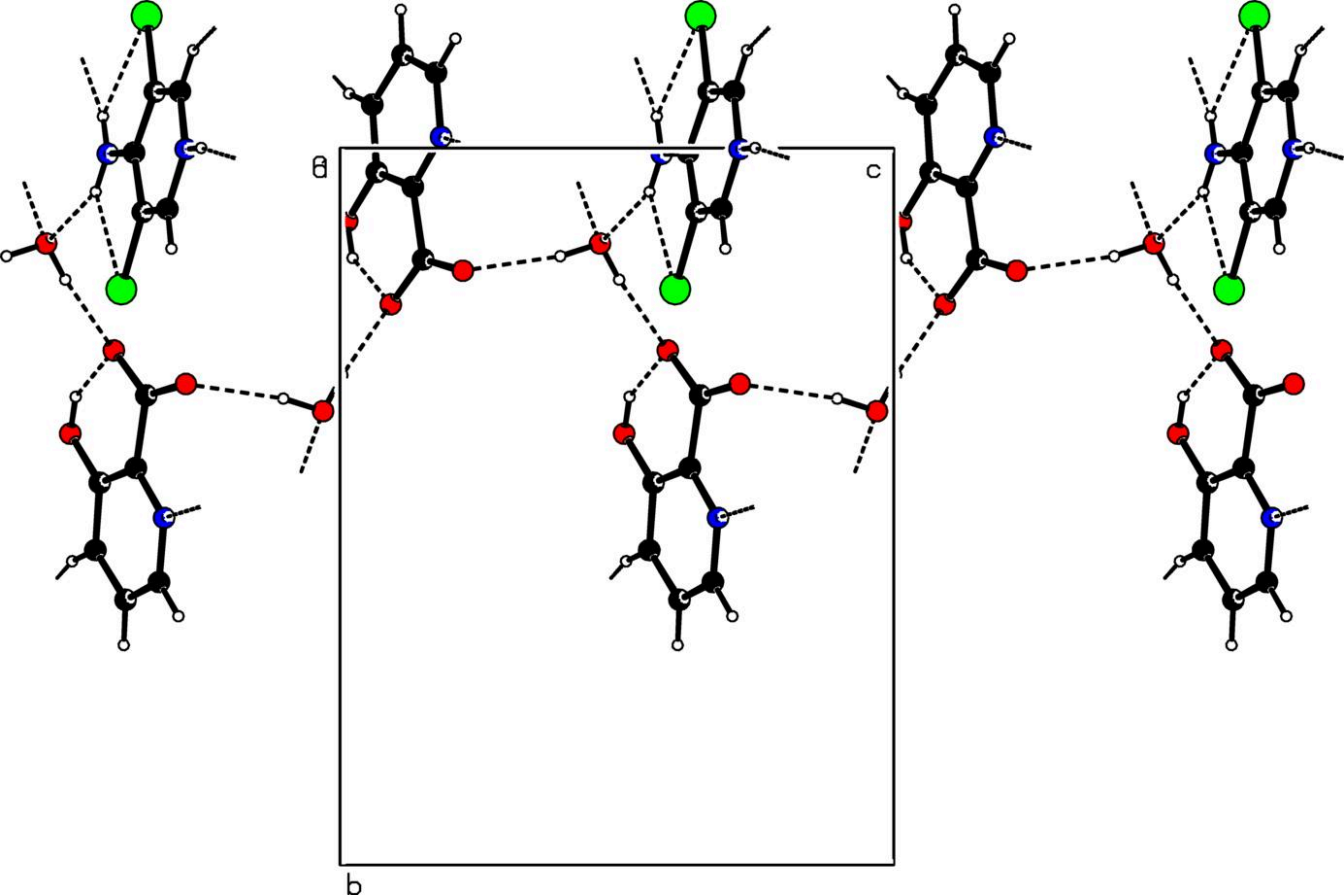
One-dimensional supra­molecular hydrogen-bonded chain mediated by water mol­ecules in the title compound.

The asymmetric unit (Fig. 1 ▸) of the title salt contains a 4-amino-3,5-di­chloro­pyridinium cation, a 3-hy­droxy picolinate anion and a water mol­ecule. The pyridinium cation is essentially planar, with a maximum deviation of 0.010 (2) Å for atom C2. A wider than normal angle [C5—N1—C1 = 120.41 (12)°] is subtended at the protonated N1 atom. In the anion, a typical intra­molecular O—H⋯O hydrogen bond, which generates an *S*(6) ring, is seen. In the extended structure, the cations, anions and water mol­ecules are connected by N—H⋯N, O—H⋯O, C—H⋯Cl, N—H⋯O and C—H⋯O hydrogen bonds (Table 1 ▸), forming a three-dimensional network (Figs. 2 ▸ and 3 ▸).

A search of the Cambridge Structural Database (Version 5.43, update November 2022; Groom *et al.*, 2016 ▸) for the 3,5-di­chloro-4-amino pyridine fragment with additional substit­uents yielded hexa­aqua­magnesium(II) bis­(4-amino-3,5,6-tri­chloro-picolinate) tetra­hydrate (CSD refcode BAWGOV; Smith *et al.*, 1981 ▸), [(4-amino-3,5-di­chloro-6-fluoro­pyridin-2-yl)­oxy]acetic acid (EZONOY; Park *et al.*, 2016 ▸), sodium picloramate hexa­hydrate (CURLIM; Smith *et al.*, 2015 ▸), guanidinium 4-amino-3,5,6-tri­chloro­picolinate (GUPICL10; Parthasarathi *et al.*, 1982 ▸), and 6-chloro-3-(tri­fluoro­meth­oxy)pyridine-2-carb­oxy­lic acid (MAFTEU; Manteau *et al.*, 2010 ▸).

## Synthesis and crystallization

A hot methanol solution of 3-hy­droxy picolinic acid (40 mg) was mixed with a hot aqueous solution of 4-amino 3,5-di­chloro pyridine (34 mg). The mixture was cooled slowly and kept at room temperature. After a few days, colourless block shaped crystals were obtained.

## Refinement

Crystal data, data collection and structure refinement details are summarized in Table 2 ▸.

## Supplementary Material

Crystal structure: contains datablock(s) global, I. DOI: 10.1107/S2414314623008210/hb4452sup1.cif

Structure factors: contains datablock(s) I. DOI: 10.1107/S2414314623008210/hb4452Isup2.hkl

CCDC reference: 2294939

Additional supporting information:  crystallographic information; 3D view; checkCIF report

## Figures and Tables

**Figure 3:**
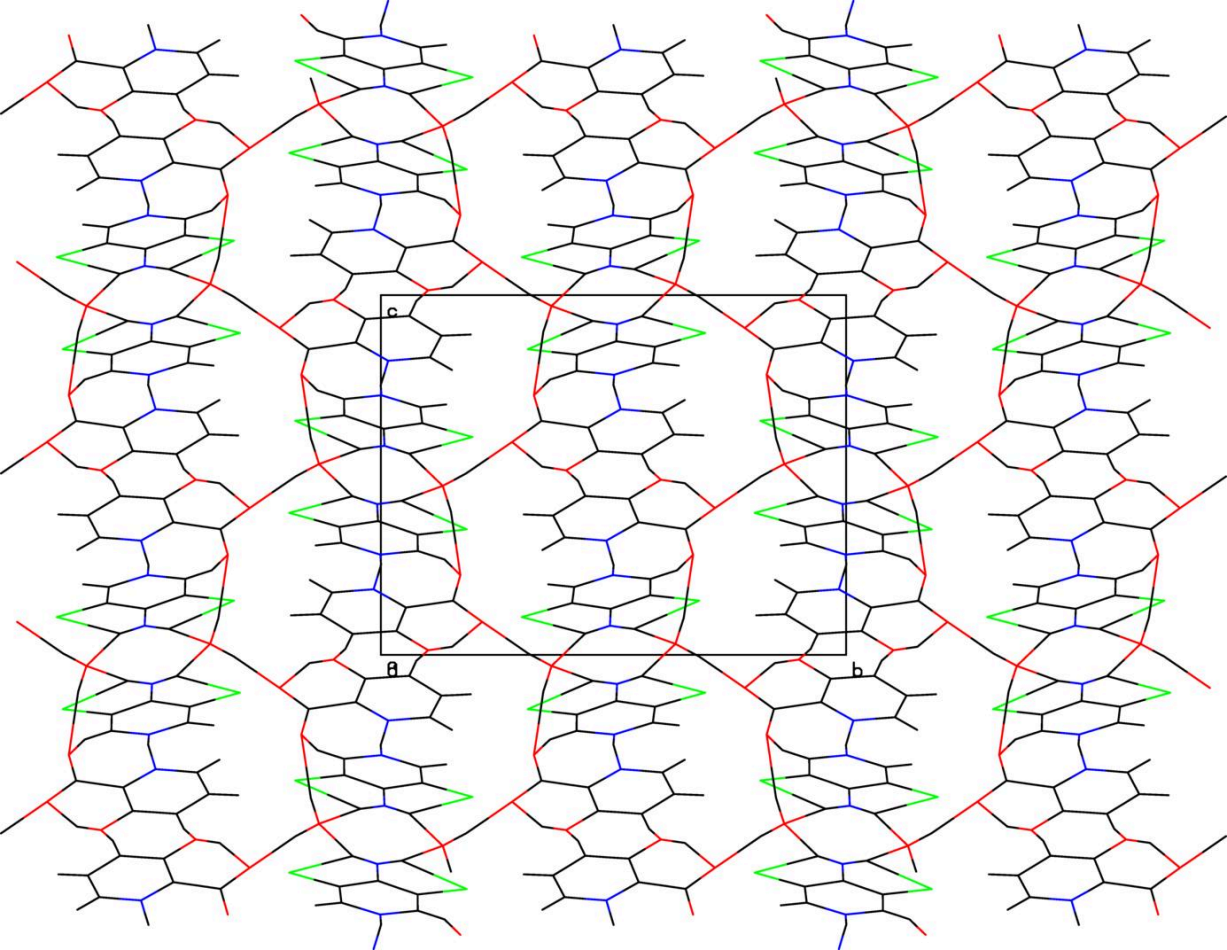
Crystal packing viewed down [100] in the title compound.

**Table 1:** Hydrogen-bond geometry (Å, °)

*D*—H⋯*A*	*D*—H	H⋯*A*	*D*⋯*A*	*D*—H⋯*A*
O3—H3⋯O2	0.82	1.79	2.5155 (17)	147
N1—H1⋯N3^i^	0.86	1.90	2.7546 (17)	171
N2—H2*A*⋯O1*W* ^ii^	0.86	2.13	2.9414 (17)	157
N2—H2*B*⋯O1*W* ^iii^	0.86	2.05	2.8269 (17)	149
O1*W*—H1*W*⋯O2	0.85	1.90	2.7442 (17)	170
O1*W*—H2*W*⋯O1^iv^	0.85	1.98	2.8181 (18)	170
C5—H5⋯O1^i^	0.93	2.31	2.9864 (18)	129
C5—H5⋯Cl2^v^	0.93	2.97	3.7363 (16)	141
C7—H7⋯O3^vi^	0.93	2.52	3.399 (2)	157

Symmetry codes: (i) 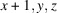 ; (ii) 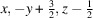 ; (iii) 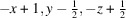 ; (iv) 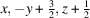 ; (v) 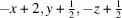 ; (vi) 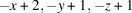 .

**Table 2:** Experimental details

Crystal data	
Chemical formula	C_5_H_5_Cl_2_N_2_^+^·C_6_H_4_NO_3_^−^·H_2_O
*M*_r_	320.13
Crystal system, space group	Monoclinic, *P*2_1_/*c*
Temperature (K)	296
*a*, *b*, *c* (Å)	8.4267 (19), 14.084 (3), 10.900 (2)
β (°)	91.953 (8)
*V* (Å^3^)	1292.9 (5)
*Z*	4
Radiation type	Mo *K*α
μ (mm^−1^)	0.52
Crystal size (mm)	0.46 × 0.32 × 0.13

Data collection	
Diffractometer	Agilent Xcalibur, Atlas, Gemini
Absorption correction	Multi-scan
*T*_min_, *T*_max_	0.819, 0.937
No. of measured, independent and observed [*I* > 2σ(*I*)] reflections	45801, 3296, 2853
*R*_int_	0.038
(sin θ/λ)_max_ (Å^−1^)	0.675

Refinement	
*R*[*F* ^2^ > 2σ(*F* ^2^)], *wR*(*F* ^2^), *S*	0.033, 0.091, 1.03
No. of reflections	3296
No. of parameters	185
H-atom treatment	H-atom parameters constrained
Δρ_max_, Δρ_min_ (e Å^−3^)	0.24, −0.35

Computer programs: *CrysAlis PRO* (Agilent, 2012 ▸), *SHELXT2018/2* (Sheldrick, 2015*a* ▸), *SHELXL2018/3* (Sheldrick, 2015*b* ▸) and *PLATON* (Spek, 2020 ▸).

## full crystallographic data

### Crystal data

**Table:**

C_5_H_5_Cl_2_N_2_^+^·C_6_H_4_NO_3_^−^·H_2_O	*F*(000) = 656
*M**_r_* = 320.13	*D*_x_ = 1.645 Mg m^−^^3^
Monoclinic, *P*2_1_/*c*	Mo *K*α radiation, λ = 0.71073 Å
*a* = 8.4267 (19) Å	Cell parameters from 3676 reflections
*b* = 14.084 (3) Å	θ = 2.5–28.8°
*c* = 10.900 (2) Å	µ = 0.52 mm^−^^1^
β = 91.953 (8)°	*T* = 296 K
*V* = 1292.9 (5) Å^3^	Plate, colourless
*Z* = 4	0.46 × 0.32 × 0.13 mm

### Data collection

**Table:**

Agilent Xcalibur, Atlas, Gemini diffractometer	2853 reflections with *I* > 2σ(*I*)
Radiation source: fine-focus sealed tube	*R*_int_ = 0.038
ω scans	θ_max_ = 28.7°, θ_min_ = 2.4°
Absorption correction: multi-scan	*h* = −11→11
*T*_min_ = 0.819, *T*_max_ = 0.937	*k* = −18→18
45801 measured reflections	*l* = −14→14
3296 independent reflections	

### Refinement

**Table:**

Refinement on *F*^2^	0 restraints
Least-squares matrix: full	Hydrogen site location: mixed
*R*[*F*^2^ > 2σ(*F*^2^)] = 0.033	H-atom parameters constrained
*wR*(*F*^2^) = 0.091	*w* = 1/[σ^2^(*F*_o_^2^) + (0.0465*P*)^2^ + 0.3959*P*] where *P* = (*F*_o_^2^ + 2*F*_c_^2^)/3
*S* = 1.03	(Δ/σ)_max_ < 0.001
3296 reflections	Δρ_max_ = 0.24 e Å^−^^3^
185 parameters	Δρ_min_ = −0.35 e Å^−^^3^

### Special details

**Table:**

Geometry. All esds (except the esd in the dihedral angle between two l.s. planes) are estimated using the full covariance matrix. The cell esds are taken into account individually in the estimation of esds in distances, angles and torsion angles; correlations between esds in cell parameters are only used when they are defined by crystal symmetry. An approximate (isotropic) treatment of cell esds is used for estimating esds involving l.s. planes.
Refinement. The water H atoms were located in a difference Fourier map and allowed to refine freely. The remaining H atoms were positioned geometrically (C—H = 0.93 and N—H = 0.86 Å) and were refined using a riding model, with *U*_iso_(H) = 1.2 *U_eq_*(C).

### Fractional atomic coordinates and isotropic or equivalent isotropic displacement parameters (Å^2^)

**Table:**

	*x*	*y*	*z*	*U*_iso_*/*U*_eq_	
Cl1	0.85851 (4)	0.68420 (3)	0.15096 (4)	0.04683 (12)	
Cl2	0.89478 (5)	0.30311 (2)	0.10521 (4)	0.04737 (12)	
O1	0.42463 (12)	0.67099 (7)	0.27823 (11)	0.0439 (3)	
O2	0.61861 (13)	0.71788 (7)	0.40818 (11)	0.0459 (3)	
O3	0.82471 (14)	0.60173 (8)	0.48694 (13)	0.0535 (3)	
H3	0.780642	0.653220	0.477230	0.080*	
N3	0.50360 (13)	0.48436 (8)	0.31752 (10)	0.0321 (2)	
N1	1.19792 (14)	0.49866 (8)	0.22147 (11)	0.0371 (3)	
H1	1.294531	0.500301	0.249479	0.045*	
N2	0.73770 (13)	0.49223 (8)	0.08094 (11)	0.0355 (3)	
H2A	0.685153	0.544264	0.072057	0.043*	
H2B	0.694906	0.439155	0.059109	0.043*	
C10	0.59379 (14)	0.55395 (9)	0.36845 (11)	0.0288 (3)	
C11	0.53872 (15)	0.65512 (9)	0.34965 (13)	0.0326 (3)	
C3	0.88438 (14)	0.49383 (9)	0.12820 (11)	0.0291 (3)	
C4	0.96128 (15)	0.57881 (9)	0.16585 (12)	0.0321 (3)	
C6	0.73510 (16)	0.53325 (10)	0.43364 (13)	0.0353 (3)	
C2	0.97853 (16)	0.41096 (9)	0.14404 (12)	0.0325 (3)	
C5	1.11412 (16)	0.57934 (10)	0.21103 (13)	0.0362 (3)	
H5	1.160941	0.636508	0.234961	0.043*	
C1	1.13192 (17)	0.41529 (10)	0.18845 (13)	0.0373 (3)	
H1A	1.191375	0.359836	0.195919	0.045*	
C9	0.55198 (17)	0.39478 (10)	0.32671 (14)	0.0388 (3)	
H9	0.488901	0.347163	0.291583	0.047*	
C8	0.69351 (18)	0.37002 (10)	0.38699 (15)	0.0428 (3)	
H8	0.725723	0.306886	0.390273	0.051*	
C7	0.78549 (17)	0.43927 (11)	0.44166 (15)	0.0431 (3)	
H7	0.880028	0.423758	0.483433	0.052*	
O1W	0.48614 (13)	0.86692 (8)	0.53048 (11)	0.0441 (3)	
H1W	0.518086	0.816660	0.495934	0.066*	
H2W	0.462944	0.849278	0.602250	0.066*	

### Atomic displacement parameters (Å^2^)

**Table:**

	*U*^11^	*U*^22^	*U*^33^	*U*^12^	*U*^13^	*U*^23^
Cl1	0.0388 (2)	0.03527 (19)	0.0658 (3)	0.00513 (13)	−0.00686 (17)	−0.01076 (16)
Cl2	0.0497 (2)	0.03070 (18)	0.0608 (3)	−0.00776 (14)	−0.01108 (18)	0.00405 (15)
O1	0.0385 (5)	0.0339 (5)	0.0585 (7)	0.0032 (4)	−0.0120 (5)	0.0042 (5)
O2	0.0429 (6)	0.0320 (5)	0.0621 (7)	−0.0008 (4)	−0.0073 (5)	−0.0116 (5)
O3	0.0416 (6)	0.0449 (6)	0.0723 (8)	−0.0023 (5)	−0.0249 (6)	−0.0072 (6)
N3	0.0294 (5)	0.0297 (5)	0.0371 (6)	0.0000 (4)	−0.0023 (4)	−0.0008 (4)
N1	0.0295 (5)	0.0419 (6)	0.0393 (6)	−0.0021 (5)	−0.0075 (5)	0.0026 (5)
N2	0.0278 (5)	0.0345 (6)	0.0438 (6)	−0.0027 (4)	−0.0051 (5)	−0.0019 (5)
C10	0.0268 (6)	0.0282 (6)	0.0313 (6)	−0.0006 (5)	0.0004 (5)	−0.0009 (5)
C11	0.0294 (6)	0.0290 (6)	0.0397 (7)	0.0010 (5)	0.0023 (5)	−0.0010 (5)
C3	0.0273 (6)	0.0342 (6)	0.0258 (6)	−0.0031 (5)	0.0007 (5)	0.0009 (5)
C4	0.0299 (6)	0.0327 (6)	0.0335 (6)	−0.0009 (5)	−0.0010 (5)	−0.0027 (5)
C6	0.0290 (6)	0.0371 (7)	0.0396 (7)	−0.0004 (5)	−0.0039 (5)	0.0003 (6)
C2	0.0340 (6)	0.0304 (6)	0.0329 (6)	−0.0047 (5)	−0.0028 (5)	0.0039 (5)
C5	0.0323 (6)	0.0387 (7)	0.0374 (7)	−0.0054 (5)	−0.0035 (5)	−0.0036 (6)
C1	0.0357 (7)	0.0363 (7)	0.0396 (7)	0.0008 (5)	−0.0048 (6)	0.0065 (6)
C9	0.0387 (7)	0.0292 (6)	0.0483 (8)	−0.0009 (5)	−0.0021 (6)	−0.0018 (6)
C8	0.0413 (8)	0.0320 (7)	0.0549 (9)	0.0084 (6)	0.0016 (7)	0.0068 (6)
C7	0.0327 (7)	0.0436 (8)	0.0526 (9)	0.0074 (6)	−0.0066 (6)	0.0075 (7)
O1W	0.0418 (6)	0.0358 (5)	0.0542 (7)	0.0069 (4)	−0.0077 (5)	−0.0016 (5)

### Geometric parameters (Å, º)

**Table:**

Cl1—C4	1.7233 (14)	C10—C11	1.5103 (18)
Cl2—C2	1.7217 (14)	C3—C4	1.4151 (17)
O1—C11	1.2366 (17)	C3—C2	1.4184 (18)
O2—C11	1.2697 (17)	C4—C5	1.3631 (18)
O3—C6	1.3446 (17)	C6—C7	1.392 (2)
O3—H3	0.8200	C2—C1	1.3661 (19)
N3—C9	1.3287 (18)	C5—H5	0.9300
N3—C10	1.3481 (16)	C1—H1A	0.9300
N1—C5	1.3406 (18)	C9—C8	1.386 (2)
N1—C1	1.3430 (18)	C9—H9	0.9300
N1—H1	0.8600	C8—C7	1.370 (2)
N2—C3	1.3229 (16)	C8—H8	0.9300
N2—H2A	0.8600	C7—H7	0.9300
N2—H2B	0.8600	O1W—H1W	0.8499
C10—C6	1.3965 (18)	O1W—H2W	0.8500
			
C6—O3—H3	109.5	O3—C6—C10	121.79 (13)
C9—N3—C10	119.47 (12)	C7—C6—C10	118.91 (13)
C5—N1—C1	120.41 (12)	C1—C2—C3	121.66 (12)
C5—N1—H1	119.8	C1—C2—Cl2	120.10 (11)
C1—N1—H1	119.8	C3—C2—Cl2	118.24 (10)
C3—N2—H2A	120.0	N1—C5—C4	120.96 (13)
C3—N2—H2B	120.0	N1—C5—H5	119.5
H2A—N2—H2B	120.0	C4—C5—H5	119.5
N3—C10—C6	121.12 (12)	N1—C1—C2	120.81 (13)
N3—C10—C11	117.62 (11)	N1—C1—H1A	119.6
C6—C10—C11	121.24 (11)	C2—C1—H1A	119.6
O1—C11—O2	125.31 (13)	N3—C9—C8	122.09 (13)
O1—C11—C10	118.99 (12)	N3—C9—H9	119.0
O2—C11—C10	115.68 (12)	C8—C9—H9	119.0
N2—C3—C4	122.67 (12)	C7—C8—C9	119.48 (13)
N2—C3—C2	123.00 (12)	C7—C8—H8	120.3
C4—C3—C2	114.33 (11)	C9—C8—H8	120.3
C5—C4—C3	121.81 (12)	C8—C7—C6	118.88 (13)
C5—C4—Cl1	119.66 (11)	C8—C7—H7	120.6
C3—C4—Cl1	118.52 (10)	C6—C7—H7	120.6
O3—C6—C7	119.29 (12)	H1W—O1W—H2W	104.5
			
C9—N3—C10—C6	−1.8 (2)	C4—C3—C2—C1	−1.97 (19)
C9—N3—C10—C11	176.65 (12)	N2—C3—C2—Cl2	−2.54 (18)
N3—C10—C11—O1	−7.84 (19)	C4—C3—C2—Cl2	178.22 (10)
C6—C10—C11—O1	170.60 (13)	C1—N1—C5—C4	−0.6 (2)
N3—C10—C11—O2	173.80 (12)	C3—C4—C5—N1	0.0 (2)
C6—C10—C11—O2	−7.76 (19)	Cl1—C4—C5—N1	−178.74 (11)
N2—C3—C4—C5	−178.05 (13)	C5—N1—C1—C2	−0.2 (2)
C2—C3—C4—C5	1.20 (19)	C3—C2—C1—N1	1.5 (2)
N2—C3—C4—Cl1	0.74 (18)	Cl2—C2—C1—N1	−178.65 (11)
C2—C3—C4—Cl1	179.99 (10)	C10—N3—C9—C8	−0.2 (2)
N3—C10—C6—O3	−178.70 (13)	N3—C9—C8—C7	1.6 (2)
C11—C10—C6—O3	2.9 (2)	C9—C8—C7—C6	−0.9 (2)
N3—C10—C6—C7	2.4 (2)	O3—C6—C7—C8	−179.94 (15)
C11—C10—C6—C7	−175.97 (13)	C10—C6—C7—C8	−1.0 (2)
N2—C3—C2—C1	177.27 (13)		

### Hydrogen-bond geometry (Å, º)

**Table:**

*D*—H···*A*	*D*—H	H···*A*	*D*···*A*	*D*—H···*A*
O3—H3···O2	0.82	1.79	2.5155 (17)	147
N1—H1···N3^i^	0.86	1.90	2.7546 (17)	171
N2—H2*A*···O1*W*^ii^	0.86	2.13	2.9414 (17)	157
N2—H2*B*···O1*W*^iii^	0.86	2.05	2.8269 (17)	149
O1*W*—H1*W*···O2	0.85	1.90	2.7442 (17)	170
O1*W*—H2*W*···O1^iv^	0.85	1.98	2.8181 (18)	170
C5—H5···O1^i^	0.93	2.31	2.9864 (18)	129
C5—H5···Cl2^v^	0.93	2.97	3.7363 (16)	141
C7—H7···O3^vi^	0.93	2.52	3.399 (2)	157

Symmetry codes: (i) *x*+1, *y*, *z*; (ii) *x*, −*y*+3/2, *z*−1/2; (iii) −*x*+1, *y*−1/2, −*z*+1/2; (iv) *x*, −*y*+3/2, *z*+1/2; (v) −*x*+2, *y*+1/2, −*z*+1/2; (vi) −*x*+2, −*y*+1, −*z*+1.
